# Possible Protective Effect of Membrane Lipid Rafts against Interleukin-1β-Mediated Anti-Proliferative Effect in INS-1 Cells

**DOI:** 10.1371/journal.pone.0102889

**Published:** 2014-07-28

**Authors:** Myriam Chentouf, Caroline Guzman, Moustafa Hamze, René Gross, Anne Dominique Lajoix, Sylvie Peraldi-Roux

**Affiliations:** 1 Université de Montpellier 1 (UM1), EA 7288, Centre de Pharmacologie et Innovation pour le Diabète (CPID), Faculté de Pharmacie, Montpellier, France; 2 Université de Montpellier 1 (UM1), EA 7288, Centre National pour la recherche scientifique (CNRS), Centre de Pharmacologie et Innovation pour le Diabète (CPID), Faculté de Pharmacie, Montpellier, France; University of Oslo, Norway

## Abstract

We recently reported that pancreatic islets from pre-diabetic rats undergo an inflammatory process in which IL-1β takes part and controls β-cell function. In the present study, using the INS-1 rat pancreatic β-cell line, we investigated the potential involvement of membrane-associated cholesterol-enriched lipid rafts in IL-1β signaling and biological effects on insulin secretion, β-cell proliferation and apoptosis. We show that, INS-1 cells exposure to increasing concentrations of IL-1β leads to a progressive inhibition of insulin release, an increase in the number of apoptotic cells and a dose-dependent decrease in pancreatic β-cell proliferation. Disruption of membrane lipid rafts markedly reduced glucose-stimulated insulin secretion but did not affect either cell apoptosis or proliferation rate, demonstrating that membrane lipid raft integrity is essential for β-cell secretory function. In the same conditions, IL-1β treatment of INS-1 cells led to a slight further decrease in insulin secretion for low concentrations of the cytokine, and a more marked one, similar to that observed in normal cells for higher concentrations. These effects occurred together with an increase in iNOS expression and surprisingly with an upregulation of tryptophane hydroxylase and protein Kinase C in membrane lipid rafts suggesting that compensatory mechanisms develop to counteract IL-1β inhibitory effects. We also demonstrate that disruption of membrane lipid rafts did not prevent cytokine-induced cell death recorded after exposure to high IL-1β concentrations. Finally, concerning cell proliferation, we bring strong evidence that membrane lipid rafts exert a protective effect against IL-1β anti-proliferative effect, possibly mediated at least partly by modifications in ERK and PKB expression/activities. Our results 1) demonstrate that IL-1β deleterious effects do not require a cholesterol-dependent plasma membrane compartmentalization of IL-1R1 signaling and 2) confer to membrane lipid rafts integrity a possible protective function that deserves to be considered in the context of inflammation and especially T2D pathogenesis.

## Introduction

Interleukin-1β (IL-1β) is a potent pro-inflammatory cytokine and a key regulator of the body's inflammatory response. IL-1β is produced after infection, injury, and antigenic challenges. It takes part in autoimmune diseases such as rheumatoid arthritis, inflammatory bowel disease, and type 1 diabetes, but also in metabolic dysregulation [1] with a disturbed secretion associated to type 2 diabetes (T2D) and impaired β-cell function [2], [3]. Indeed in T2D, metabolic stress activates the innate immune system, resulting in a chronic inflammatory state marked by increased cytokines, increased islet-associated macrophages, and β-cell apoptosis [4]–[6]. Surprisingly, IL1-R1 is highly expressed in β-cells [7] which is in line with their high sensitivity to IL-1β.

There is growing evidence that IL-1β plays a dual role in insulin secretion as well as in β-cell mass regulation. Furthermore, it has also been suggested that rather than being directly cytotoxic, IL-1β may drive tissue inflammation that impacts on both β-cell functional mass and insulin sensitivity in T2D [8]. Indeed, several studies point to beneficial effects of low concentrations of IL-1β on β-cell proliferation, apoptosis, and secretory function in rat and human islets [9], [10], whereas high IL-1β levels are known to impair insulin secretion, to decrease β-cell proliferation and to induce apoptosis [11].

A major step in IL-1β signaling is the activation of the transcription factor NFκB. IL-1R1 dimerization is an early event in IL-1β signaling after ligand binding [12], [13]. This event initiates binding of MyD88 to the Toll-IL-1R1 domains within the cytoplasmic tail of IL-1R1. Subsequently, multiple receptor/ligand pairs are endocytosed into a specialized signaling endosome. Then, the downstream recruitment of the IL-1R1 effectors TRAF6, IRAK1, and other MAP kinases lead to the phosphorylation of IKK. IKK activation in turn triggers the release of NFκB from IκB, allowing nuclear translocation of NFκB to transcriptionally activate downstream target genes including a large number of cytokines or proteins, apoptotic factors, anti-apoptotic factors, and other transcription factors.

IL-1R1 is constitutively present in membrane lipid raft fractions-regardless of IL-1β whereas MyD88 is found in lipid rafts after IL-1β stimulation [14]. This suggests that IL-1R1 activation and IL-1β signaling are dependent on membrane lipid rafts. These plasma membrane microdomains, enriched in cholesterol and glycosphingolipids, have been identified as platforms for receptor signaling and constitute important integrators of signal events and intracellular trafficking.

In this respect, defects in insulin signaling due to membrane lipid raft alterations have been suggested to play an important role in the pathogenesis of insulin resistance [15]. Indeed, disruption of caveolae in cultured cells by cholesterol extraction with methyl β-cyclodextrin (MβCD) results in the progressive inhibition of tyrosine phosphorylation of IRS-1, as well as a reduced activation of glucose transport in response to insulin [16]. Furthermore, elevated blood cholesterol in obese individuals is harmful to human health, and is related to the development of T2D. In addition, insulin secretion in primary β-cells is highly sensitive to changes in plasma membrane cholesterol [17].Therefore, cholesterol homeostasis in pancreatic β-cells is critical for maintaining appropriate signaling pathways and a normal β-cell function.

In the present study, we examined the effect of IL-1β on β-cell function through membrane lipid rafts signaling. To mimic pancreatic β-cells inflammatory process observed during the development of T2D, we used INS-1 cells incubated with low and high IL-1β concentrations. We bring experimental evidence for a possible role but limited of membrane lipid rafts in IL-1β signaling steps leading to the inhibition of insulin secretion and question their involvement in β-cell death. We investigated the expression level of a panel of proteins and their association to membrane lipid rafts after IL-1β stimulation of INS-1 cells; we could demonstrate that a number of proteins involved in apoptosis, cell proliferation and insulin secretion are overexpressed or recruited to membrane lipid rafts. Furthermore, we show that the disruption of membrane lipid rafts only very partially affects and is even able to reinforce the deleterious effect of IL-1β on INS-1 cells function. Therefore, our findings bring evidence that IL-1β signaling and its biological effects occur independently of cholesterol-related plasma membrane compartmentalization; the pathways involved and responsible for IL-1β pathogenic effects are independent of membrane lipid rafts. However, our study also strongly suggests that lipid rafts integrity affords some protection against IL-1β deleterious effects.

## Materials and Methods

### Materials

INS-1 rat insulinoma β cells used in the study have been obtained by CB Wollheim [18]. For immunofluorescence studies, we used rabbit anti-cytokines antibodies against IL-1 β (H-153), anti-cytokine receptor antibodies against IL-1R1 (M-20) (Santa Cruz Biotechnology, Santa Cruz, CA). Fluorescein isothiocyanate (FITC)-conjugated anti-rabbit (Vector Laboratories, Burlingame, CA) was used as secondary antibody. Ganglioside M1 (GM1) detection has been performed using Alexa 488 Fluor conjugated-Cholera toxin B subunit (Invitrogen). Western blot experiments were performed with rabbit anti-IL1-R1 (H-150) (Santa Cruz Biotechnology) and horseradish peroxidase–conjugated anti-rabbit antibody (Sigma-Aldrich) was used to detect the signal. We used peroxidase-conjugated cholera toxin B subunit (Invitrogen) for dot-blot experiments. For apoptosis detection, rabbit polyclonal anti-annexin V antibody (Abcam, Cambridge, UK) was used at 5 mg/ml. Recombinant rat IL-1β and IL-1Ra were purchased from R&D Systems (Minneapolis, MN).

### Brij 98-DRM raft isolation

A total of 1.10^8^ INS-1 cells were treated with IL-1β. After washing in 160 mM PBS (pH 7.4), cell lysis was performed at 37°C for 30 min in 1% Brij 98 detergent diluted in TNE buffer (25 mM Tris-HCl (pH 7.5); 150 mM NaCl; and 5 mM EDTA) containing 1 mg/ml enzyme inhibitors (complete EDTA-free mixture of anti-proteases; Roche, Meylan, France). Cell lysates were mixed with an equal volume of 80% sucrose in TNE plus inhibitors, overlaid with 6.5 ml of 30% and 3.5 ml of 5% sucrose in TNE plus inhibitors, and then centrifuged at 200,000 g for 20 hours [19], [20]. From the top of the gradients, 9 1-ml fractions were collected on ice and numbered from 1 to 9. Protein in each fraction was quantified by using the micro Bradford Protein Assay kit (Pierce, Rockford, USA).

### Dot-blot analysis of ganglioside M1-enriched Brij 98 DRM

A nitrocellulose membrane (Hybond ECL; Amersham Pharmacia Biotech) was spotted with 2 µg from each gradient fraction. The membrane was blocked for 1 hour at room temperature with 5% semi skimmed milk in PBS containing 0.1% Tween 20 (PBS-T). Ganglioside M1 (GM1) detection in Brij 98 DRM was performed by adding a 1/1000 solution of peroxidase-conjugated cholera toxin B subunit and incubating for 1 hour at room temperature. After washing in PBS-T, binding was revealed using the ECL Western Blotting Detection kit (Amersham Pharmacia Biotech).

### Western-blotting

Membrane lipid raft and non raft proteins (40 mg) were fractionated on a 12% polyacrylamide gel and transferred to a nitrocellulose membrane. After blocking with 5% dried skim milk or BSA, filters were then incubated overnight with anti-IL-1R1 (1/100 Santa Cruz Biotechnology). After three washings, membranes were incubated with a horseradish peroxidase–conjugated anti-rabbit antibody (diluted 1∶3000; Sigma-Aldrich). Immunoreactivity was detected using an enhanced chemiluminescence reaction (Amersham Biosciences, Little Chalfont, U.K) and analyzed with the chemiluminescence imager (Chemismart 5000, Fisher Bioblock, France).

### Immunofluorescence studies on β-cells

β-cells were grown during 72 hours and seeded on LabTech chamber slide system (Sigma), previously coated with poly-L-lysine at 0.1 mg/ml (Sigma). Cells were cultured in RPMI medium containing 10% of fetal calf serum (FCS), 50 U/mL penicillin, 50 mg/mL streptomycin, 2 mmol/L glutamine (Life technology, France), 1 M hepes, 100 mM sodium pyruvate, 50 mM 2-mercaptoethanol. Then, cells were washed with PBS (pH 7.4) containing CaCl2 and MgCl2, fixed with 3% paraformaldehyde for 30 min, permeabilized 5 min in 0.1% Triton X-100 and quenched with 50 mM NH4Cl for 10 min. After two washings with PBS, the slides were saturated with 2% BSA-Gelatine solution and then incubated with a primary antibody (anti-rabbit IL-1β or IL-1R1 antibody) diluted at 1/50 overnight at 4°C. After three washings, cells were incubated for 1 hour with FITC-conjugated antibodies. For detection of lipid rafts, we used Alexa 488 Fluor conjugated-Cholera toxin B subunit diluted 1/1000 on non permeabilized INS-1 cells incubated for 1 hour. After three additional washings, cells were covered with citifluor (Citifluor, U.K.) and observed with an upright fluorescence microscope (Leica DMRA) or with confocal microscope (Leica SD5-SMD) using the facilities of RIO imagery platform (Montpellier, France). Negative controls were performed by incubating the cells with irrelevant antibody (anti rabbit-IgG), diluted 1/100 and by incubation with the secondary antibody alone.

### Antibody Arrays

Panorama antibody microarray containing 224 different antibodies spotted in duplicate on nitrocellulose-coated glass slides was purchased from Sigma-Aldrich. Protein extracts (1 mg/ml) from INS-1 rat insulinoma β cells membrane lipid rafts and non rafts (without or with IL-1β stimulation) were labeled with Cy3 and Cy5 (Amersham Biosciences, Buckingham, UK) as described by the manufacturer (Sigma Aldrich, Steinheim, Germany). Samples labeled with a dye/protein molar ratio 2 were applied to the antibody microarray in Array Incubation Buffer (Sigma) and incubated for 45 min protected from light with gentle shaking. The array was then washed three times with 5 ml of Washing Buffer (Sigma) and air-dried. Cy3 and Cy5 signals were read on the Gene Pix Pro 4.0 (MDS Analytical Technologies). Each experiment was carried out twice and analyzed for both Cy3 and Cy5 signals. Proteins whose expression was found down or upregulated by 2 fold or more versus control were considered as significant.

### Insulin secretion by INS-1 Cells

Cells were cultured for 2 days in the absence or in the presence of increasing IL-1β concentrations (0.01, 0.1, 1, 10 and 100 ng/ml). At the end of the 48 hours exposure period, cells were then washed, deprived in glucose during 1 hour and incubated in the presence of 2.8; 5.6; 8.3 and 16 mM glucose. Cells supernatant fractions were then collected and insulin content was extracted with acid/alcohol mixture (1.5%/75%). Quantification of insulin content and insulin present in INS-1 cells supernatants were performed using HTRF assay (Cisbio, Marcoule, France). Insulin secretion data were normalized for insulin contents as ratios of insulin secretion to insulin content and then expressed as percentages of insulin release in the presence of 16 mM glucose alone or in the absence of treatment.

To inhibit IL-1β effect on insulin secretion, INS-1 cells were pre-incubated with 500 ng/ml IL-1Ra during 1 hour before IL-1β treatment. Insulin secretion was measured in the presence of 8.3 mM glucose as the stimulating condition.

### Apoptosis assay

Apoptosis was detected using annexin V antibody. Annexin V is a Ca2+-dependent phospholipid-binding protein with a high affinity for phosphatidylserine (PS). In normal cells, PS is located on the cytoplasmic surface of the cell membrane. In apoptotic cells, PS is translocated from the inner to the outer surface of the cell membrane. Annexin V labelling can identify apoptotic cells by binding to PS exposed on the outer leaflet. INS-1 cells cultured in LabTech chamber slide (as described) were treated with vehicle or with IL-1β (0.01; 0.1; 1; 10; 100 ng/ml) for 48 hours and then washed once with ice-cold PBS, immunostained with rabbit polyclonal anti-annexin V antibody (Abcam) and then fixed with 3% paraformaldehyde for 30 min. Cells were washed again and analyzed by confocal microscopy with excitation at 488 nm (green, annexin V).

Early and late apoptosis of rat pancreatic β cells treated by IL-1β were detected using FACS analysis. Cells were detached from the culture flasks by Hepes-EDTA buffer (Hepes 10 mM, EDTA 3 mg/ml), rinsed and pelleted (5 min, 1000 rpm, 4°C) in PBS with 2% FCS (FACS buffer). The cells (∼10^6^) were incubated with 200 µl of FACS buffer containing 10 µg/ml of rabbit anti-annexin V FITC antibody and propidium iodide for 90 min at 4°C. After washings with PBS, the cells were analyzed (10000 events) on an EPICS cytofluorometer (Beckman-Coulter, Fullerton, CA).

### Proliferation assay

INS-1 cells growth was assessed after 5 days of culture using the cell bromodeoxyuridine (BrdU) proliferation kit (Roche) according to the manufacturer's instructions. INS-1 cells were cultured in triplicate in 96-well culture microplates at 2×10^4^ cells per well (Techno plastic products, Trasadingen, Switzerland) in 200 µl of culture medium in the absence or presence of IL-1β. Microplates were then incubated for 2 days at 37°C in a wet atmosphere containing 5% CO_2_. Bromodeoxyuridine 1/100 dilution was then added, and microplates incubated for an additional 24 hours. Bromodeoxyuridine incorporation was measured using a horseradish peroxidase-conjugated anti-BrdU antibody and *o*-phenylenediamine (100 µl per well) as substrate. The colorimetric reaction was stopped by addition of 50 µl of 4 n sulphuric acid per well and absorbance measured at 490 nm.

### INS-1 cells pretreatment by inhibitors

1.10^8^ cells were pre-incubated with 10 mM MβCD, disolved in PBS for 30 min at 37°C to extract cholesterol. Cells were washed with PBS to remove inhibitors as described above. Absence of inhibitor toxicity was confirmed by trypan blue exclusion in pre-treated and untreated cells.

### Statistical analysis

All data were expressed as the means ± SEM of 3 experiments performed independently. Differences were analyzed either by Student-*t*-test or by 2-way ANOVA analysis. p<0.05 was taken as threshold value for statistical significance.

## Results

### INS-1 cell line expresses IL-1R1 in lipid rafts

**Figure 1:**
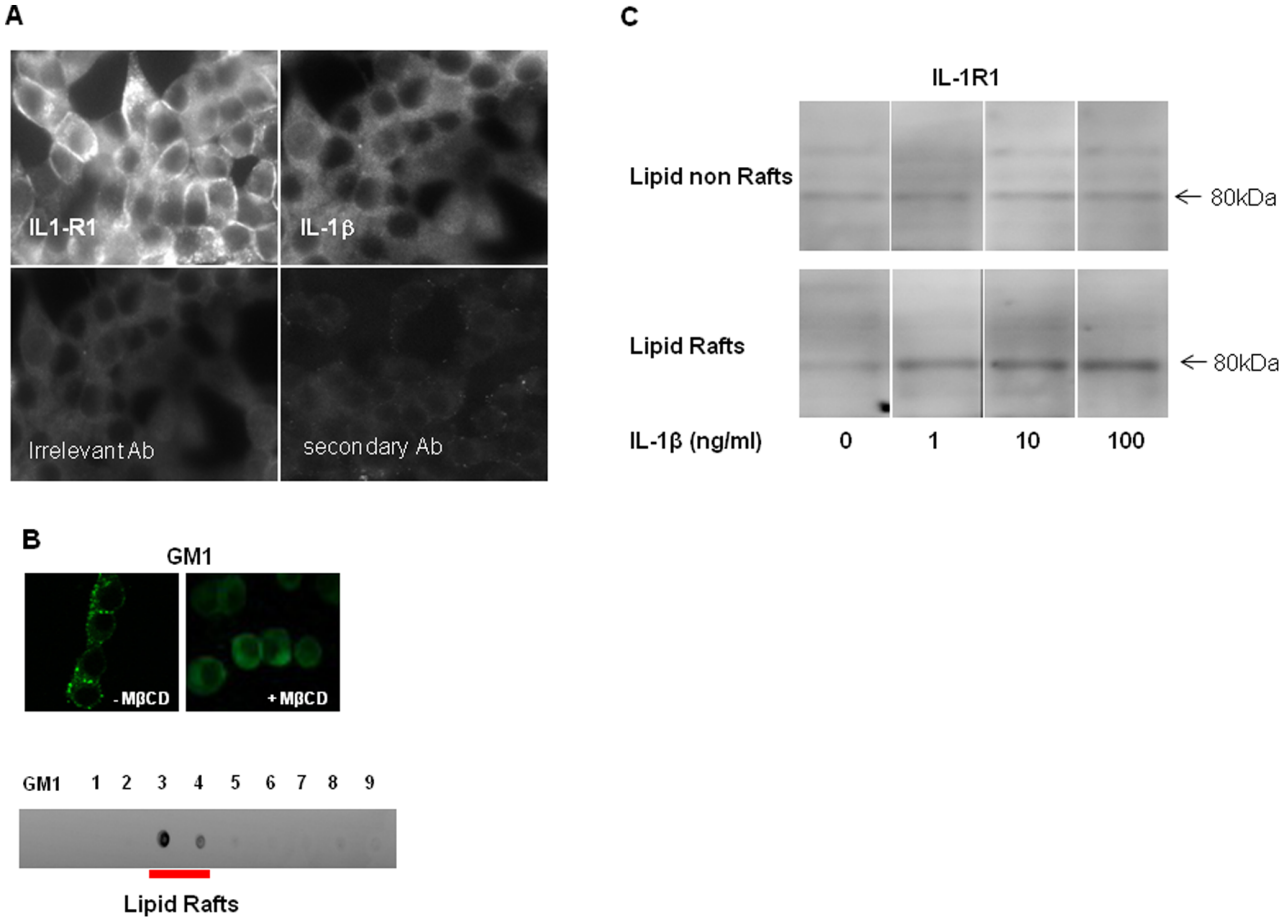
INS-1 cell line expresses IL-1R1 in lipid rafts. (A) Immunofluorescence studies in the INS-1 cell line; Immunostaining of receptor IL1-R1 and cytokine IL1-β. Irrelevant rabbit IgG and secondary antibody were used as negative controls. (B) Characterization of lipid raft fractions by Ganglioside M1 detection after isolation on sucrose gradient; Dot Blot characterization of Brij 98-extracted fractions. INS-1 cells were lysed with Brij 98 detergent at 37°C and separated into fractions by sucrose density gradient. Membrane rafts are present in fractions 3 and 4, as indicated by GM1 expression. Membrane lipid rafts localization in the INS-1 cell line by GM1 detection using immunofluorescence, without and with MβCD treatment. Results are representative of three independent experiments. (C) Western blots of IL-1R1 expression in lipid-rafts and non-rafts fractions. Forty micrograms of protein extracts from membrane lipid rafts- and non-lipid rafts were run on SDS-PAGE and blotted with anti Il-1R1 antibody.

We first characterized the presence of IL-1R1 on pancreatic INS-1 cells and isolated membrane lipid rafts. We found IL-1R1 expressed in INS-1 cells and associated with the β-cell surface and insulin granules. No immunostaining could be observed with the anti-IL-1β antibody (Fig. 1A).

To localize the cellular distribution of lipid rafts in the INS-1 cell line, we performed confocal immunofluorescence microscopy using Ganglioside M1 (GM1) as a marker (Fig. 1B). Immunolabelling points to a marked expression in the pancreatic β-cell line. GM1 appears strongly expressed all over the cell plasma membrane, but was also found present in the intracellular space. Interestingly a punctuated labeling was observed at the cell-cell contact. After treatment with MβCD no GM1 immunostainning could be observed demonstrating the disruption of membrane lipid rafts.

After membrane lipid raft isolation procedure, two main fractions, 3 and 4, proved to contain membrane lipid rafts with a larger amount in fraction 3. The two fractions were pooled and used to characterize the presence of IL-1R1 by Western blotting. As we hypothesized that the level of IL-1R1 occurring in membrane lipid rafts could change after stimulation, we evaluated the amounts of the receptor associated with isolated membrane lipid rafts prior to and follow IL1-β stimulation. As expected, IL-1R1 is expressed in lipid rafts fraction in the absence of IL-1β, demonstrating that IL-1R1 is constitutively present in- and associated to membrane lipid rafts on INS-1 cells (Fig. 1C). In IL-1β treated cells, the expression of IL-1R1 increased in membrane lipid rafts but remained unchanged in non-lipid raft fractions.

### Effect of IL-1β on β-cell function: insulin secretion, apoptose and proliferation

**Figure 2:**
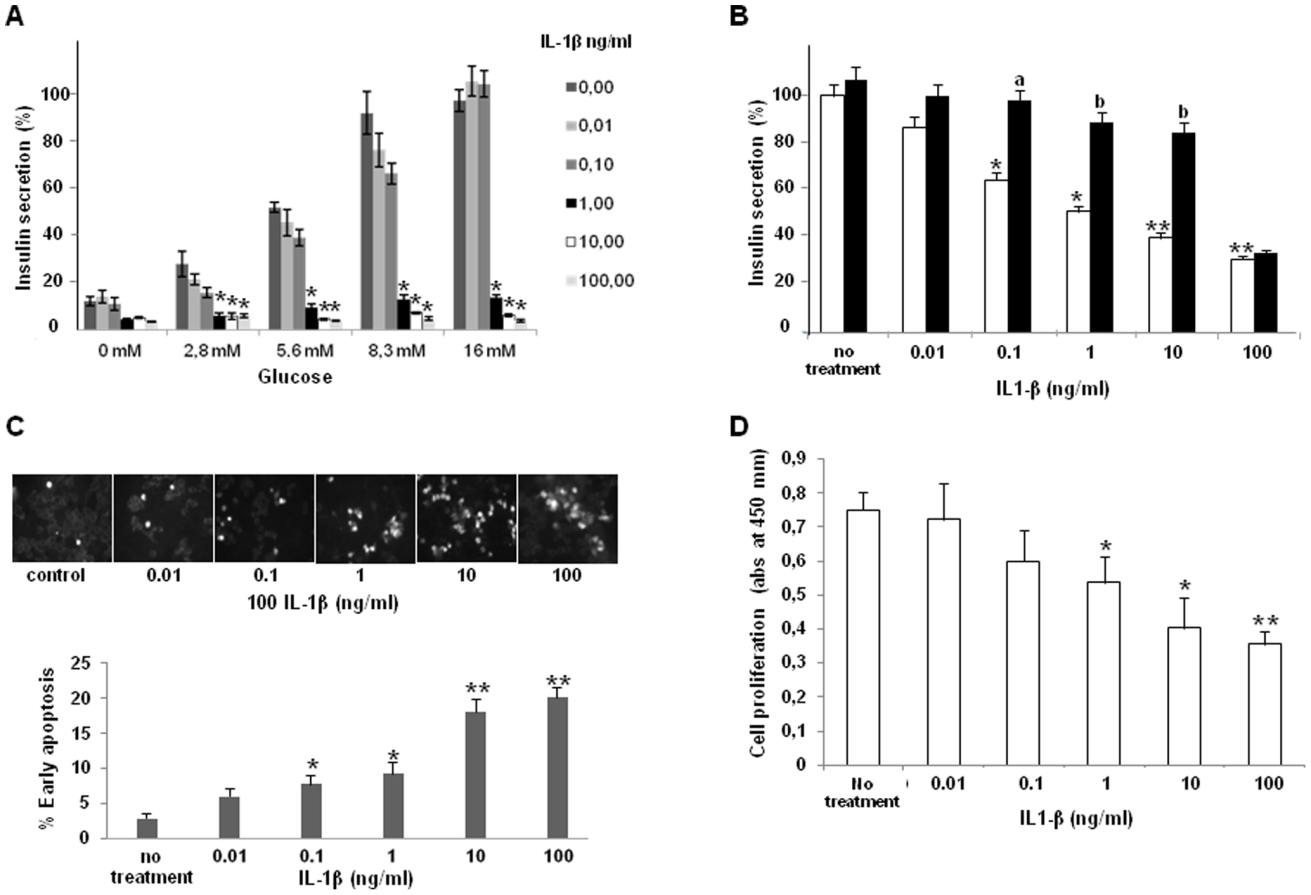
Effect of IL-1β on β-cell function: insulin secretion, apoptose and proliferation. (A) Insulin secretion assay on INS-1 cells cultured in the presence of increasing IL-1β concentrations for 2 days. At the end of the exposure time, cells were washed, deprived in glucose during 1 hour and incubated in the presence of 0, 2.8, 5.6, 8.3 and 16 mM glucose. Cells supernatant fractions were collected and insulin content extracted with acid/alcohol mixture. Insulin present in culture supernatants and insulin content was quantified using HTRF assay. (B) Insulin secretion with IL-1β treatment (White column), and after pre-treatment of INS-1 cells with 500 ng/ml of IL1 receptor 1 antagonist (IL1-RA) to inhibit IL1-β effect on secretion (Black column). Insulin has been quantified by HTRF. Fig (2A-2B): All data represent insulin release normalized for insulin contents and are expressed as percentages of insulin secretion recorded in the presence of 16 mM glucose alone; they are all the result of 3 independent experiments, with each experimental condition performed in triplicate. (C) Annexin V labeling of apoptotic INS-1 cells after treatment by increased concentrations of IL-1β: Ins-1 cells were analyzed by confocal microscopy (pictures are representative of 3 independent experiments) and flow cytometry (see Fig 3B). (D) Cell proliferation test by BrdU incorporation in INS-1 cells after IL-1β treatment; controls are cells not exposed to the cytokine. Data represent means of 3 independent experiments with each experimental condition performed in triplicates. *: p<0.05 and **: p<0.01 vs cells incubated in the absence of IL-1 β; a: p<0.05 and b: p<0.01 vs in the absence of antagonist.

The presence of IL-1R1 in INS-1 β-cells and its constitutive expression in membrane lipid rafts prompted us to study the effect of IL-1β on insulin release, apoptosis and proliferation. INS-1 cells were cultured in the presence of increasing IL-1β concentrations for 2 days (Fig. 2A). Exposure to 0.01 ng/ml and 0.1 ng/ml of IL-1β did not modify glucose induced insulin secretion (5.6 to 16 mM glucose). In contrast, exposure to higher concentrations, 1 to 100 ng/ml IL-1β resulted into a progressive inhibition of insulin release reaching about 80% for 100 ng/ml. IL-1Ra, the antagonist of IL-1R1, when used at the concentration of 500 ng/ml, significantly counteracted the inhibitory effect of 0.1 to 10 ng/ml IL-1 β on glucose-induced insulin secretion, but did not modify the effect of the cytokine at 100 ng/ml (Fig. 2B).

**Figure 3:**
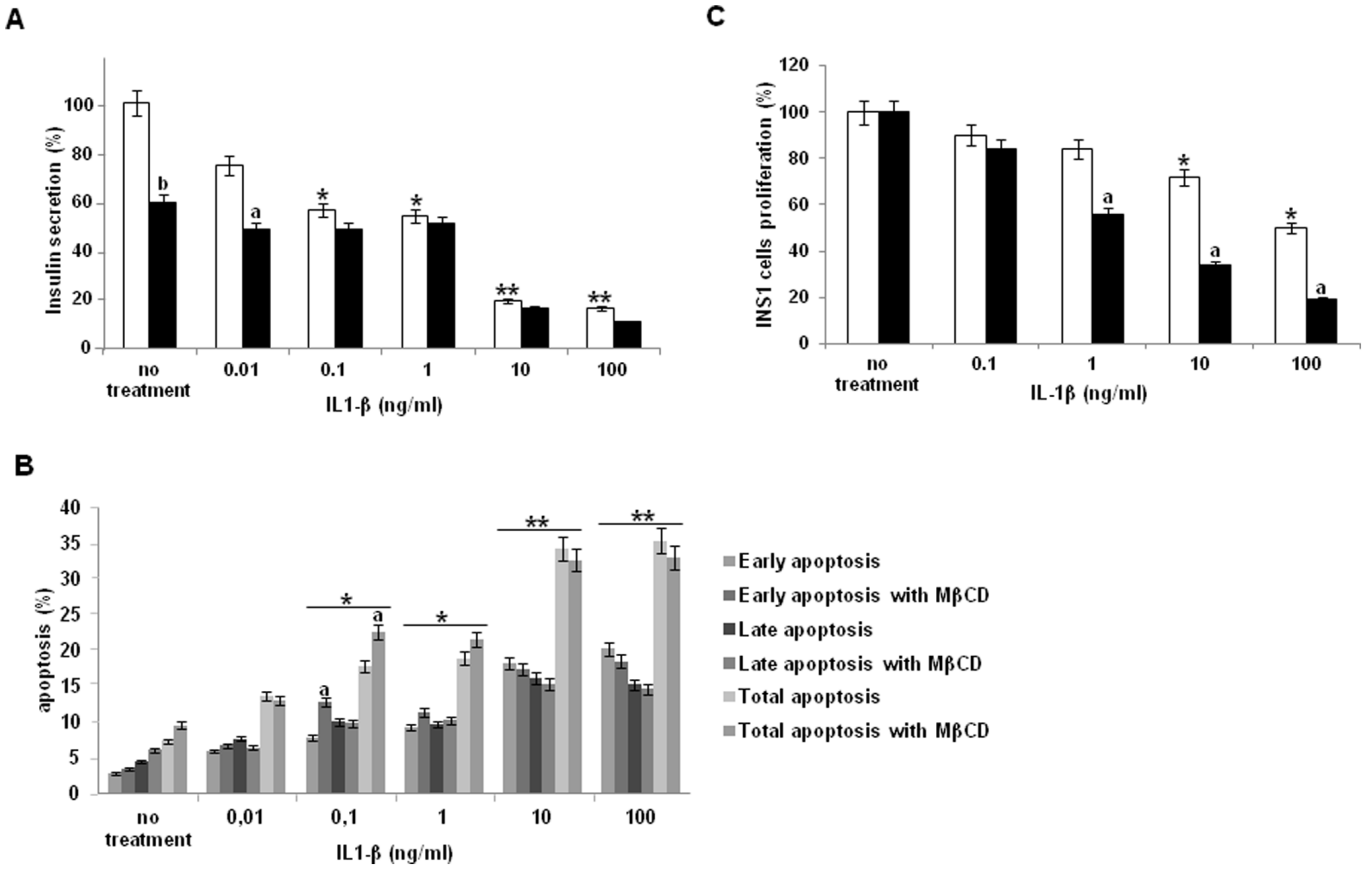
Lipid rafts integrity and IL-1β effects on insulin secretion, apoptosis and cell proliferation. (A) Insulin secretion after MβCD treatment to induce cholesterol depletion and disturb lipid raft domains. INS-1 cells were stimulated by increasing concentrations of IL1-β and insulin secretion was quantified by HTRF assay. INS-1 cell without MβCD treatment (White column), and INS-1 cell pre-treated by MβCD (Black column). All data represent insulin release normalized for insulin contents and are expressed as percentages of insulin secretion recorded in the absence of any treatment. (B) INS-1 apoptosis. INS-1 cells were stimulated by IL-1β with or without MβCD pre-treatment, and induction of apoptosis was determined using Annexin V FITC/propidium iodide staining in flow cytometry. (C) Analysis of INS-1 proliferation using BrdU incorporation, with or without MβCD treatment and after IL-1β stimulation. INS-1 cell without MβCD treatment (White column), and INS-1 cell pre-treated by MβCD (Black column). Fig 3 (A-B-C) Data are means of 3 experiments with each experimental condition performed as triplicates. *: p<0.05 and **: p<0.01 vs non- IL-1β pretreated cells; a: p<0.05 and b: p<0.01 vs non- MβCD treated cells.

We also measured the effect of IL-1β on islet-cell viability using confocal microscopy after annexin staining. After the 2-day culture in the presence of low concentrations of IL-1β (0.01 to 0.1 ng/ml), only small levels of positively stained apoptotic cells could be detected (Fig. 2C). However in the presence of higher IL-1β concentrations, (10 to 100 ng/ml), the number of apoptotic cells observed by confocal microscopy clearly increased (Fig. 2C). We also measured the effect of IL-1β on islet-cell viability using Flow cytometry after annexin and IP staining. After the 2-day culture in the presence of low concentrations of IL-1β (0.1 and 1 ng/ml), only low amounts of dead INS-1 cells could be detected (17.8 and 18.9% vs 7.3% in non-treated control cells). However in the presence of higher IL-1β concentrations, (10 to 100 ng/ml), the number of total apoptotic cells observed by flow cytometry clearly increased (34.3% and 35.4% respectively) (Fig. 3B). Finally, INS-1 cells proliferation was evaluated using BrdU assay. The anti-proliferative effect of IL-1β appeared to be clearly IL-1β dose-dependent. A significant decrease in pancreatic β-cells proliferation was observed for 10 and 100 ng/ml, with a maximum (50%) at 100 ng/ml IL-1β (Fig. 2D) suggesting that the decrease in INS-1 cells secretory function observed after chronic treatments with high concentrations of IL-1β could result from the combination of direct effects on insulin release but also on cell proliferation and apoptosis.

### Membrane lipid rafts integrity play a role for insulin secretion

Removal of plasma membrane cholesterol reduces exoctytosis in neuroendocrine cells [21], [22] but until now, there is no consensus concerning insulin-secreting cells. We investigated whether structural integrity of membrane lipid rafts is required for IL-1β effect on insulin secretion using MβCD, a cholesterol-binding agent that depletes plasma membrane cholesterol content [23] and disrupts membrane lipid rafts.

We measured glucose-stimulated insulin secretion after chronic exposure to 0.01 to 100 ng/ml IL-1β in intact INS-1 cells and after disruption of membrane lipid rafts. Depleting INS-1 cell membrane cholesterol with MβCD drastically reduced glucose-stimulated insulin secretion by 40% (Fig. 3A), which demonstrates that membrane lipid raft integrity is an essential feature for β-cell secretory function. The reduction of insulin release was obtained using 10 mM MβCD, and in the presence of 8.3 mM glucose. To preclude the interplay of possible effects of IL-1β on insulin synthesis, we normalized INS-1 cells insulin release for total insulin content (see Materials and Methods).

To determine whether IL-1β inhibitory effect still operates after membrane cholesterol depletion, we investigated the effect of an exposure to increasing concentrations of the cytokine in MβCD treated cells. After disruption of membrane lipid rafts (leading to a 40% decrease of control glucose-induced insulin secretion), incubation of INS-1 cells with low to moderate IL-1β concentrations resulted into only minor inhibitory effects of the cytokine; indeed, insulin release appears to be further decreased by 18 to 14% versus 25 to 45% in intact cells. In contrast, in the presence of 10 and 100 ng/ml IL-1β, insulin secretion values were further decreased to values similar in MβCD-treated and non-treated cells. These data suggest that for high IL1-β concentrations, non lipid rafts IL1-R1 receptors operate and probably account for the drastic inhibitory effect on insulin secretion.

### The apoptotic effect of IL-1β on INS-1 cells is not dependant of membrane lipid rafts integrity

The role of membrane lipid rafts on INS-1 cell viability upon chronic IL-1β treatment was determined and early and late apoptosis or necrosis were evaluated after respectively annexin- and IP stainings. Exposure of INS-1 cells to low (0.01-1 ng/ml) IL-1β concentrations for 2 days moderately affected early and late apoptosis versus non-exposed controls with total cell apoptosis rates ranging between 13.0 and 18.9% (Fig. 3B). However, higher IL-1β concentrations (10 and 100 ng/ml) led to significant increases in cell death, with respectively 32.7 and 33.1% dead cells. Disruption of membrane lipid rafts by MβCD did not prevent cytokine-induced cell death recorded after exposure to high IL-1β concentrations, suggesting that IL-1 β-induced apoptosis occurs independently of membrane lipid rafts.

### Membrane lipid rafts integrity protects INS-1 cells against Il-1β anti-proliferative effect

Finally, to analyze the role of membrane lipid rafts in IL-1 β anti-proliferative action, we compared the effects of a chronic exposure to increasing cytokine concentrations in MβCD pretreated and non-pretreated INS-1 cells. Disruption of membrane lipid rafts integrity did not modify INS-1cells basal proliferation level. Interestingly, MβCD treatment appeared able to significantly reinforce IL-1β anti-proliferative effect (Fig. 3C). Indeed, membrane lipid rafts disruption increased the cytokine effect by 6, 28.5, 38.5 and 37% for respectively 0.1, 1, 10 and 100 ng/ml IL-1β. These data strongly suggest that membrane lipid rafts exert a protection against IL-1β anti-proliferative effect and that a mechanism related to the integrity of their organization counteracts the deleterious effect of the cytokine on cell proliferation.

### IL-1β induces expression of proteins mainly in membrane lipid rafts

There is growing evidence that membrane lipid rafts are membrane micro-domains involved in the compartmentalization of a number of cellular processes including signal transduction and membrane trafficking. Therefore, we analyzed the effect of IL-1β on the expression level of various proteins in lipid rafts- and non-rafts fractions using low and high concentration of cytokine (0.1 ng/ml to 100 ng/ml). We used an antibody array, with more than two hundred distinct antibodies printed at high-density on a glass microscope slide. The proteins studied are involved in apoptosis, cell cycle, cytoskeleton, nuclear signaling, neurobiology, and signal transduction. Three types of modifications in the expression pattern of different proteins were observed upon exposure to IL-1β. (1) An overexpression of proteins involved in apoptosis (Activated Caspase 3), cellular stress (CHOP-10, inducible isoform of Nitric Oxide Synthase (iNOS)), and signal transduction (MAP kinases ERK1/2, PKC, Phospho Focal adhesion protein (FAK)) in membrane lipid rafts in the absence of any change of their expression level versus controls in the non-raft membrane fractions. (2) A decreased expression level in membrane lipids rafts for Pyk2 with no change in non-lipid raft fractions. (3) A recruitment from non- membrane lipid rafts to lipid rafts for PKB/Akt. Indeed, the increased expression in membrane lipid rafts occurred simultaneously with a decreased expression in non-lipid raft fractions especially at the high (100 ng/ml) IL-1β concentration. Moreover, CHOP-10, PKB/Akt and PKC were weakly expressed in membrane lipid rafts under control conditions were found induced/recruited in membrane lipid rafts after exposure of INS-1 cells to IL-1β (Table 1).

**Table 1:** IL1-β effects on protein expression in lipid raft- and non-lipid raft fractions.

	Lipid Rafts			Non Lipid Rafts		
*IL-1β (ng/ml)*	*0.1*	*1.0*	*100.*	*0.1*	*1.*	*100.*
Activated caspase 3	x 1.65	x 1.29	x 2.24	x 1.10	x 1.01	x 1.08
GADD 153 (CHOP-10)	x 7.51	x 6.12	x 10.01	x 1.09	x 1.04	x 1.01
Cdk6	x 1.69	x 2.10	x 1.28	x 1.17	x 1.36	x 0.65
i-NOS	x 1.88	x 2.21	x 2.41	x 1.26	x 1.26	x 1.26
Trp Hydroxylase	x 1.04	x 21.23	x 18.48	x 0.85	x 0.89	x 0.80
FAK pY577	x 0.42	x 0.39	x 0.31	x 5.95	x 6.63	x 7.6
FAK pY397	x 1.43	x 1.91	x 9.70	x 0.99	x 0.65	x 1.31
MAP Kinase (Erk1+Erk2)	x 1.92	x 1.75	x 2.07	x 1.04	x 0.84	x 1.02
MAP Kinase pYT	x 5.22	x 6.53	x 6.07	x 0.93	x 0.99	x 0.91
MAP Kinase pY	x 2.94	x 3.52	x 3.59	x 0.82	x 0.92	x 1.26
MPKK2	x 3.51	x 2.47	x 3.84	x 1.21	x 0.94	x 0.88
PKB/AKT	x 1.88	x 3.14	x 2.33	x 1.06	x 0.82	x 0.67
PKC β	x 1.40	x 0.91	x 2.07	x 1.07	x 0.76	x 1.28
PKCγ	x 7.09	x 2.44	x 5.02	x 1.43	x 0.66	x 1.64
Pyk2 Phospho (pY579)	x 0.28	x 0.35	x 0.23	x 1.43	x 0.91	x 0.96
Pyk2 Phospho (pY580)	x 0.32	x 0.34	x 0.28	x 1.26	x 0.72	x 0.83

Changes in the expression pattern of proteins involved in signal transduction, apoptosis, and cell cycle were examined by antibody Array. Protein extracts (1 mg/ml) from INS-1 cells membrane lipid rafts and non lipid rafts, treated or not by IL1-β, were labeled with Cy3 and Cy5. Equal amounts of labelled proteins were incubated with antibodies doted on nitrocellulose-coated glass slides. The level of protein expression was quantified and the most representative changes are summarized on this Table.

Up or down regulated membrane lipid raft and non lipid raft proteins after IL-1β treatment are presented in relative values recorded for the same proteins expressed in raft and non raft fraction in control condition (no IL-1β treatment). A 2-fold increase or decrease (bold) taken as cut-off value (positive or negative) is considered as significant.

### IL-1β increases Caspase-3, CHOP-10 and iNOS expression in membrane lipid rafts

Among proteins mediating apoptosis [24], caspases, a family of ubiquitous proteases, play a central role. Caspase-3 is a key protein of the apoptotic pathway [25]. It is activated by proteolytic cleavage into 19 and 17 kDa subunits. Our data point to an increase by more than 2 fold (2.2) of activated caspase 3 into lipid raft fraction after a 48 h exposure to 100 ng/ml IL-1β, suggesting that caspases-mediated IL-1β apoptotic effect on INS-1 cells requires high concentrations of the cytokine. On the other hand, apoptosis induced by cellular stress could appear at lower IL-1β concentrations. The pro-apoptotic endothelial reticulum (ER) stress marker CHOP-10 was found weakly expressed in control cells and no change occurred in non-lipid raft fractions upon exposure to IL-1β. In contrast, the cytokine appeared able to strongly and concentration-dependently increase CHOP-10 expression by 7.5 to 10.0 fold versus controls in lipid raft fractions. Interestingly, it has been described that CHOP-10 expression is induced by inflammatory cytokines via nitric oxide signaling [26]. Our results also point to a more than 2 fold increase of iNOS expression in membrane lipid rafts which is in line with CHOP-10 over-expression.

### IL-1β and changes in the expression of membrane lipid raft proteins involved in the control of cell proliferation

IL-1β also provoked significant modifications in expression pattern of proteins involved in cell proliferation, in membrane lipid rafts. MAP kinases protein expression was increased by 1.9 to 2.1 fold (for ERK1/2) after exposure of cells to the cytokine, whereas the amounts of their phosphorylated form (pERK1/pERK2) reached 5.2 to 6.1 fold higher levels than in control cells. However, it must be noticed that the ratio between activated and non-activated forms of ERK1/ERK2 was maximal (x3.7) for 1 ng/ml IL-1β and then moderately decreased for higher concentrations. PI3 kinase and its downstream effector protein kinase (PKB/Akt) are also known as key regulators of cell survival. In our experiments, we found that PKB followed a pattern of expression similar to that recorded for MAP kinases: a 1.88- and 3.11 fold increases for respectively 0.1 and 1 ng/ml IL-1β versus 2.33 fold at 100 ng/ml IL-1β (Table 1).

Taken together, both sets of experiments suggest that ERK and PKB might be two components activated at low IL-1β concentrations, with PKB ensuring a possible protective effect on INS-1 cells survival. In addition, expression of Grb2, an adaptor protein also involved in cell proliferation, was found unchanged in membrane lipid rafts but decreased by about 1/3 in the non raft fractions, which agrees with the reduction in cell proliferation upon exposure to high IL-1β concentrations. Finally, two non-receptor kinases have been found to be inversely modified. Our data point to strong modifications in the amounts of the phosphorylated forms of the kinase upon exposure to IL-1β. First FAK pY-397 is strongly increased in lipid rafts after exposure to high (100 ng/ml) IL-1β concentrations. Second, FAK pY-577 the phosphorylated form, with maximal kinase activity, is markedly enhanced for already low to moderate IL-1β concentrations in the non-lipid raft fractions. In addition, the two phosphorylated forms of Pyk2, a tyrosine kinase structurally related to FAK were also found drastically reduced after IL-1β treatment.

### Il-1β and changes in the expression of proteins involved in insulin secretion in lipid raft and non- raft fractions

The high amounts of iNOS are well known to inhibit insulin secretion and to induce pancreatic β-cell destruction [27]. In our experimental conditions, IL-1β (0.1-100 ng/ml) induced a progressive 1.88 to 2.41 fold increase in iNOS expression in membrane lipid rafts which probably plays a central in the deleterious effects of the cytokine.

5-HT plays a central role as a substrate for protein serotonylation that regulates granule transport in pancreatic β-cells [28]. We found that IL-1β strongly increased the expression of tryptophane hydroxylase (TH), the enzyme responsible for 5-HT synthesis. TH expression was increased by 17- and 21 fold for 0.1 and 1 ng/ml IL-1β in membrane lipid rafts whereas no change occurred in the non- raft fractions. Likewise exposure of INS-1 cells to IL-1β also increased expression of Protein Kinase C (PKCβ); the enzyme has previously been proposed to operate in the stimulus-secretion coupling and stimulating effects of IL-1β on insulin secretion. The stronger increase (x7.09 versus controls) was obtained in presence of the cytokine at the lowest concentration. Our data suggest that both TH and PKC increased expression in membrane lipid rafts could make up compensatory mechanisms aimed at counteracting IL-1β inhibitory effects.

## Discussion

There is growing evidence that pro-inflammatory cytokines are implicated in the dysfunction of pancreatic β-cell in T2D, but the signalling pathways involved remain poorly characterized. Activation of signalling transduction pathways at the level of plasma membrane lipid rafts has been reported to be the first critical step required for cytokines to initiate their biological effects on cells [29]. Furthermore, it has been shown that T2D is associated with lipid deposits in tissues and aberrant accumulation of ceramide [30] which could disturb membrane raft structural integrity, cause delocalization of raft proteins, change proteins interactions and consequently modify signal transduction. In this context, the aim of you study was to determine to what extent membrane lipid rafts contribute to IL-1 β effect on β-cell secretory function, -apoptosis and -proliferation using the INS-1 rat pancreatic cell line.

Concerning insulin secretion, although disruption of pancreatic β-cell lipid rafts has been reported to increase exocytotic events [31], most studies point to an inhibitory effect found to be associated to a disruption and dispersion of syntaxin 1 clusters on the plasma membrane [32] and migration of SNAP-25 from the membrane to the cytosol [17]. Our data confirm that membrane cholesterol is an essential factor in the regulation of insulin secretion. Indeed, disturbance of lipid rafts using MβCD leads to a decrease of glucose-stimulated insulin secretion suggesting that insulin release in INS-1 cells is dependent on cholesterol-sensitive processes confined to the plasma membrane. Such observations might be of pathological relevance; indeed, sub-chronic exposure of pancreatic β-cells to high glucose has been shown to induce a decrease in membrane cholesterol, disturbing membrane lipid rafts stability and resulting into a loss of syntaxin 1A from granule docking sites and inhibition of insulin secretion [33].

Since the early studies by Spinas and coworkers [34], IL-1β is well known to exert a bimodal effect on insulin secretion depending on the concentration, duration of exposure of the cells to the cytokine and glucose concentration; low concentrations of IL-1β stimulate insulin release in rat islets and high concentrations of cytokine decrease insulin release. Our data show that a 48-hour exposure of INS-1 cells to increasing concentrations of the cytokine, concentration-dependently inhibits insulin secretion. We also bring evidence that cholesterol removal and IL-1β do not exert additive effects. The cytokine induced only a weak further decrease in insulin secretion after MβCD treatment suggesting that part of IL-1β effect occurs via mechanisms underpinned by membrane lipid rafts integrity. In contrast, the effect of higher (10 and 100 ng/ml) concentrations of the cytokine is independent of membrane lipid rafts and probably mediated by binding to- and activation of IL-1R1 receptors either insensitive to cholesterol removal or present on the non-lipid raft part of plasma membrane. Such an effect very likely occurs at 10 ng/ml as IL-1β inhibitory effect is for a great part antagonized by IL-1Ra, in non-MβCD treated cells. This is not the case for the highest (100 ng/ml) concentration which questions the mechanism(s) involved. It must however be mentioned that the maximal inhibitory effect is almost achieved at 10 ng/ml.

IL-1β released by T lymphocytes and activated macrophages as well as by pancreatic β cells has been implicated as an effector molecule that both inhibits insulin secretion and provokes β-cell destruction with NO mediating these deleterious effects [27]. In this respect, the increased expression of iNOS we found in lipid raft fractions probably plays an important role, all the more so as disruption of membrane lipid rafts has been shown to markedly reduce IL-1β-induced gene expression of iNOS and NO release [35]. It must be emphasized that increased iNOS expression has been reported in the mildly diabetic GK rat [36] and in T2D human pancreatic islets [37]. In our study, the increase in membrane lipid rafts iNOS is probably involved in IL-1β-induced inhibition of insulin secretion for low to moderate concentrations of the cytokine and operates via mechanisms depending on- and integrated by membrane lipid rafts. However, at higher concentrations, IL-1β effects not affected by cholesterol removal are lipid rafts- and therefore very likely NO-independent and are probably mediated by different transduction pathways activated in different plasma membrane domains.

Of great interest is our observation that PKC expression is strongly increased in lipid raft fractions upon exposure to IL-1β. PKC is a kinase known to modulate Ca^2+^ triggered exocytosis from β-cells [38], [39] and thus hormonal control of glucose-induced insulin secretion. The increased expression of the kinase in membrane lipid rafts at 0.1 and 1 ng/ml could afford a protective effect aimed at counteracting the inhibition of insulin secretion. Such a mechanism appears less pronounced at 100 ng/ml, which is in line with the reduced expression in the non-lipid raft fractions. A similar attention should be paid to the drastic increase in TH expression. The enzyme responsible for 5-HT synthesis is thereby implicated in insulin granules transport via serotonylation of GTPases within pancreatic β-cells [28]. Such an effect downstream of Ca^2+^-signaling could, in concert with the increase in PKC expression, participate in a mechanism aimed at challenging IL-1β deleterious effects. In this respect, it must also be mentioned that FAK, the activity of which was strongly increased after exposure to high IL-1β concentrations in lipid rafts and more consistently in non-raft fractions, is critical for β-cell secretory function [40] and that glucose-stimulated phosphorylation of FAK is involved in the full development of glucose-induced secretion [41].

In our study we could confirm the pro-apoptotic and anti-proliferative effects of IL-1β. Concerning β-cell survival, IL-1β is known to induce β-cell death via the activation of gene networks controlled by the transcription factors NFkB and STAT-1. Activation of NF-kappaB results into NO production and depleted endoplasmic reticulum (ER) Ca^2+^ stores. β -cell death then occurs through activation of MAP kinases via triggering of ER stress and the release of mitochondrial death signals [4]. The increased expression levels of iNOS, CHOP-10 and actived caspase 3 we found in lipid raft fractions support the concept that membrane lipid rafts are involved in IL-1β biological effects and agree with the increase amount of IL-1R1 after exposure of INS-1 cells to the cytokine. However, two important issues deserve to be addressed. First, if IL-1β was able to inhibit cell proliferation by more than 50%, maximal apoptotic effect only reached about 35%. Second, IL-1β effects were not reduced by membrane cholesterol removal; they were even found amplified. The modest apoptotic effect of IL-1β could be due to our experimental conditions; in this respect, recent studies have shown that mild ER stress predisposes and sensitizes β-cells to IL-1β pro-apoptotic effects [42]. The limitation of the latter could also, as for insulin secretion, result from the existence of protective mechanisms that compensate in part for the deleterious effects. Their persistence after membrane lipid raft disruption demonstrates that IL-1β also operates via membrane lipid raft-independent transduction pathways, which also agrees with the ability for the cytokine to induce apoptosis independently of NO and the presence of IL-1R1 in the non-lipid raft part of β-cell membrane. Their amplification and especially for anti-proliferative effects after membrane cholesterol removal, argues for the existence of protective mechanisms located in lipid rafts.

Our data point to a marked increase in the activity of ERK1/2 pathway in lipid raft fractions. The molecular mechanisms that define the conditions for ERK-mediated control of cell survival remain poorly understood. ERK1/2 is generally known for exerting pro-apoptotic functions, however activation of ERK1/2 has also been reported to promote cell survival [43] and there is evidence that phosphorylation of Bim and/or Bad by ERK1/2 through multiples mechanisms can contribute to reduce the sensitivity of cells to apoptosis and promote cell proliferation [44]. Our data do not allow us to conclude whether ERK1/2 activity results into deleterious or benefic effects; however, the increases in apoptosis and proliferation after membrane lipid raft disruption point to beneficial ones and deleterious effects, if any, should be of limited importance.

In this respect, it must also be mentioned that we observed a strong increase of the amounts of the activated form of Focal Adhesion Kinase (FAK). Recent studies have shown that FAK deletion results into reduced pancreatic β-cell viability and proliferation [40]. This non-receptor kinase is known to interact with other signaling pathways to promote cell migration, proliferation and survival and to inhibit apoptosis [45]. It is noteworthy that FAK has been shown to regulate cyclin D1 expression, an effect mediated by its activation of the ERK pathway [46] and largely dependent on phosphorylation of Tyr-397 [25]. pY-397 has also been identified as a binding site for PI3-kinase, well-known to deliver proliferative and anti-apoptotic signals. Likewise, of interest are also the enhanced levels of FAK pY577 in non-lipid raft fractions which might at least partly contribute to the relative resistance of INS-1 cells to the pro-apoptotic and anti-proliferative effects of IL-1β. At the opposite, we observed a strong decrease in the amounts of the activated forms of a FAK structurally related tyrosine kinase, Pyk2, which unlike FAK [47] has been shown to inhibit G (1) to S phase transition [48] and to induce apoptosis both in vitro [49] and in vivo [50]. On the same line we also found an increased expression of the serine/threonine protein kinase PKB, which upon activation stimulates cell cycle progression, survival, metabolism and migration through phosphorylation of many physiological substrates [51]. Taken together, our observation concerning the increases in PKB expression, FAK activities as well as the decrease in Pyk2 activity, strongly suggest that membrane lipid rafts underpin and integrate a number of mechanism aimed at compensating IL-1β deleterious effects on INS-1 cell survival and proliferation. The increased apoptotic effect of IL-1β at low and moderate concentration after membrane lipid raft disruption and especially the more marked decrease in cell proliferation in the same conditions, testify that these compensating mechanisms do operate.

In conclusion, we demonstrated that the transduction pathways involved in IL-1β pathogenic effects do not require a cholesterol dependent compartmentalization of β-cell plasma membrane; they operate independently of membrane lipid rafts. In contrast, our observations confer to membrane lipid rafts integrity a possible protective function that deserves to be considered in the context of inflammation and especially T2D pathogenesis.
